# Application of ultrasound elastography and radiomic for predicting central cervical lymph node metastasis in papillary thyroid microcarcinoma

**DOI:** 10.3389/fonc.2024.1354288

**Published:** 2024-05-10

**Authors:** Liuxi Wu, Yasu Zhou, Lu Li, Wenting Ma, Hongyan Deng, Xinhua Ye

**Affiliations:** Department of Ultrasound, The First Affiliated Hospital of Nanjing Medical University, Nanjing, China

**Keywords:** ultrasound, elastography, radiomic, papillary thyroid microcarcinoma, central cervical lymph node

## Abstract

**Objective:**

This study aims to combine ultrasound (US) elastography (USE) and radiomic to predict central cervical lymph node metastasis (CLNM) in patients with papillary thyroid microcarcinoma (PTMC).

**Methods:**

A total of 204 patients with 204 thyroid nodules who were confirmed with PTMC and treated in our hospital were enrolled and randomly assigned to the training set (n = 142) and the validation set (n = 62). US features, USE (gender, shape, echogenic foci, thyroid imaging reporting and data system (TIRADS) category, and elasticity score), and radiomic signature were employed to build three models. A nomogram was plotted for the combined model, and decision curve analysis was applied for clinical use.

**Results:**

The combined model (USE and radiomic) showed optimal diagnostic performance in both training (AUC = 0.868) and validation sets (AUC = 0.857), outperforming other models.

**Conclusion:**

The combined model based on USE and radiomic showed a superior performance in the prediction of CLNM of patients with PTMC, covering the shortage of low specificity of conventional US in detecting CLNM.

## Introduction

Papillary thyroid microcarcinoma (PTMC), which is defined as a papillary thyroid carcinoma (PTC) tumor of 1 cm or less in size, has shown an increased occurrence in result of the widespread use of high-resolution ultrasonography (US) and fine-needle aspiration biopsy (FNAB) (1–3). Despite the excellent prognosis of PTMC, central lymph node metastasis (CLNM) is common among patients with PTMC, and the occurrence can be as high as 64.1%, which implies poor overall survival (4, 5). According to the current guideline (1), PTMCs with aggressive features, such as clinical node metastasis, distant metastasis, and invasive symptoms to the recurrent laryngeal nerve or trachea, were supposed to complete therapeutic central compartment lymph node dissection (CLND) during initial thyroidectomy. Low-risk PTMCs such as clinical node-negative (cN0) PTMCs, showing no clinical evidence of CLNM on US or other imaging modalities preoperatively, have other options besides immediate surgery, including active surveillance and thyroidectomy without CLND (6). However, The preoperative detection rate of CLNM remains quite low, and CLNM proved by histopathological examination is as high as 31% to 60.9% in PTMCs (7, 8). This situation makes accurate evaluation of lymph node status preoperatively particularly important. On one hand, CLND may bring unnecessary complications and economic burdens in pathological lymph node-negative PTMC (9). On the other hand, simple thyroidectomy may lead to disease recurrence, and second surgery is more difficult for CLND (10). In the era of precision medicine, it remains controversial whether CLND should be taken in PTMCs, making it crucial that predictors of CLNM should be screened out and accurate evaluation need to be performed preoperatively.

Ultrasound elastography (USE), an imaging technology sensitive to tissue stiffness, which was first described in the 1990s, is more objective as a tool to evaluate tissue hardness than clinical palpation. USE can be classified into strain elastography (SE) and shear wave elastography (SWE) by the measured physical quantity (11). Recent study reported that SWE showed a great value to differentiate metastatic and benign lymph nodes in PTCs (12). By using SWE to evaluate the stiffness of PTC nodules, higher elasticity values were found to be associated with pathologic central or lateral LN metastasis, improving the sensitivity for prediction of CLNM from 28% to 45% compared with gray-scale US alone (13). According to the different pattern of SE image, a scoring system, that is, elasticity score (ES), class 1 to 4, was used to describe the stiffness of tissue (14). SE has been reported to be useful for predicting extrathyroidal extension (15, 16), which should be helpful in the diagnosis and the evaluation of possible recurrence of PTMC. SE has also proved to be an effective addition to US to better predict malignancy and describe thyroid nodules (17). However, to our knowledge, there are few literatures evaluating the application of ES in the prediction of CLNM of PTMC until now.

Radiomic is emerging as a promising tool that quantitatively extracts high-throughput features and converts medical images into mineable data such as the information of pathology, biomarkers, and genomics, improving the diagnostic, predictive, and prognostic accuracy by applying it in clinical-decision support system (18, 19). In recent years, radiomic has been widely used in tumor research, obtaining satisfactory achievements. Previous study showed that CT-based radiomic signature performed well in the prediction of lymph node metastasis in PTCs and that radiomic features from the enhancement phase played a leading role in the process (20). Several studies have proved that radiomic analysis based on ultrasound has great value for noninvasively predicting cervical lymph node metastasis (CLNM) in PTCs (18, 21). We hypothesized that radiomic may provide more information about PTMCs preoperatively, and it should be able to help identify patients with high-risk PTMC and make more suitable medical plan.

In this study, we aimed to analyze the performance of USE and radiomic in the prediction of CLNM of PTMC and visualize the probability of risk factors in order to facilitate designing an optimal treatment strategy.

## Materials and methods

### Patients

This retrospective study was approved by the Ethics Committee and performed in accordance with the Declaration of Helsinki (2022-SR-512). Considering that it is a retrospective study, the requirement for informed consent was waived. From November 2021 to October 2022, patients confirmed with PTMC and treated in our hospital were involved in this study. Inclusion criteria are as follows: (1) patients with histopathologically confirmed PTMC; (2) patients who underwent primary thyroid surgery; (3) pathology of central cervical lymph lode was confirmed by surgery or fine needle aspiration biopsy (FNAB); and (4) patients whose preoperative ultrasound examination was performed 1 week before surgery and full data of clinical characteristics and ultrasound image can be achieved. Exclusion criteria are as follows: (1) patients received preoperative interventional therapies; (2) patients with incomplete clinical data; and (3) patients whose ultrasound image did not meet the requirement for radiomic analysis. Finally, 204 patients with a total of 204 thyroid nodules were enrolled and randomly assigned to training set (n = 142) and validation set (n = 62) at a 7:3 ratio. In each set, the patients were divided into two groups based on the pathological results of CLND or FNAB. Clinical variables including gender, age, and BRAF ^V600E^ mutation status (wild type/mutant type) were recorded.

### US image acquisition

All patients underwent a preoperative US examination on the thyroid nodule by a Samsung XR80A ultrasound machine, with a 3- to 12-MHz linear probe. The US features of thyroid nodule were observed and recorded by two US physicians with more than 10 years’ experience in thyroid US examination, including capsular contact (positive/negative), aspect ratio (<1/=1/>1), shape (regular/irregular), echogenicity (very hypoechoic/hypoechoic/isoechoic or hyperechoic), echogenic foci (none/punctate echogenic foci/macrocalcifications), blood flow (none/hypervascular/mild or moderate), TIRADS category (3/4a/4b/4c/5), using thyroid imaging reporting and data system developed by Kwak et al. (22), and ES. The ES was ranked from 1 to 4, which is equal to the elasticity from soft to hard. When disagreements appeared between the physicians, the third senior US physician reviewed the features and made the final decision. The US image chosen for radiomic analysis should meet the following requirements: (1) containing as much malignant features as possible of the thyroid nodule in the transverse section; (2) stored in digital imaging and communications in medicine (DICOM) format and without any marks; and (3) captured by the same settings about gain, depth and frequency.

### Region-of-interest segmentation and radiomic feature extraction

After being exported from the ultrasound instrument in DICOM format, the US images in the maximum transection area were segmented by an ultrasound expert (more than 5 years of experience) using open-source software (ITK-SNAP 3.8.0; http://www.itksnap.org) to generate a region of interest (ROI) containing the thyroid nodule. Finally, a total of 464 radiomic features were extracted from the US images, consisting of nine shape features, 90 first-order features, and 365 texture features.

### Radiomic feature selection and signature calculation

The reproducibility of radiomic feature extraction was evaluated based on the inter and intra-operator coefficient (ICC). Three weeks after the radiomic feature extraction, the same ultrasound expert randomly selected 30 lesions from the training set to draw ROI again, and another radiologist with more than 5 years of experience repeated the work independently. An independent samples *t*-test was used to evaluate the inter and intra-operator differences. ICC > 0.75 was suggestive of a good agreement.

The least absolute shrinkage selection operator (LASSO) with 10-fold cross validation was used to selected candidate radiomic features for a radscore calculating. The radscore is calculating according to the following formula:


Radscore=β0+β1χ1+β2χ2+⋯+βnχn

where β0 is the constant term in the regression, βi is logistic regression coefficient, and χ*i* is the value of the selected features.

### Construction of three different models and model performance assessment

Univariate analysis was used to analyze the impact factor of clinical variables and US characteristics. Model 1 was constructed with the US features of *p<* 0.05 plus ES of the training set. Model 2 was constructed with the optimal features selected by LASSO logistic regression in the training set. Model 3 is based on the combination of selected clinical, US characteristics, ES, and radscore.

The established three models were validated using validation set. The receiver operating curve (ROC) was used to evaluate the discriminant ability of three models by calculating the area under curve (AUC), sensitivity, and specificity of them.

### Visualization of model 3

For the sake of precision medicine, a nomogram, convenient for clinical decision, was plotted. Decision curve analysis (DCA) was employed to determine the clinical usefulness by quantifying the net benefits in both training set and validation set. Calibration curve was applied to evaluate the correction in both sets.

### Statistical analysis

Statistical analyses including univariate analysis and binary logistic regression that was applied to build models were conducted by SPSS software (version 26.0). Chi-square test (χ^2^) or Fisher exact test were used to compare differences for categorical variables. Normal distribution decided whether the independent sample *t* test or the Mann-Whitney U-test to be used for continuous variables analysis. R software (version 3.6.1, http://www.r-project.org) was used for radiomic features analysis, radscore construction, and model evaluation. Two-sided *p<* 0.05 was assumed to indicate statistical significance.

## Results

### Patient characteristics

**Figure 1:**
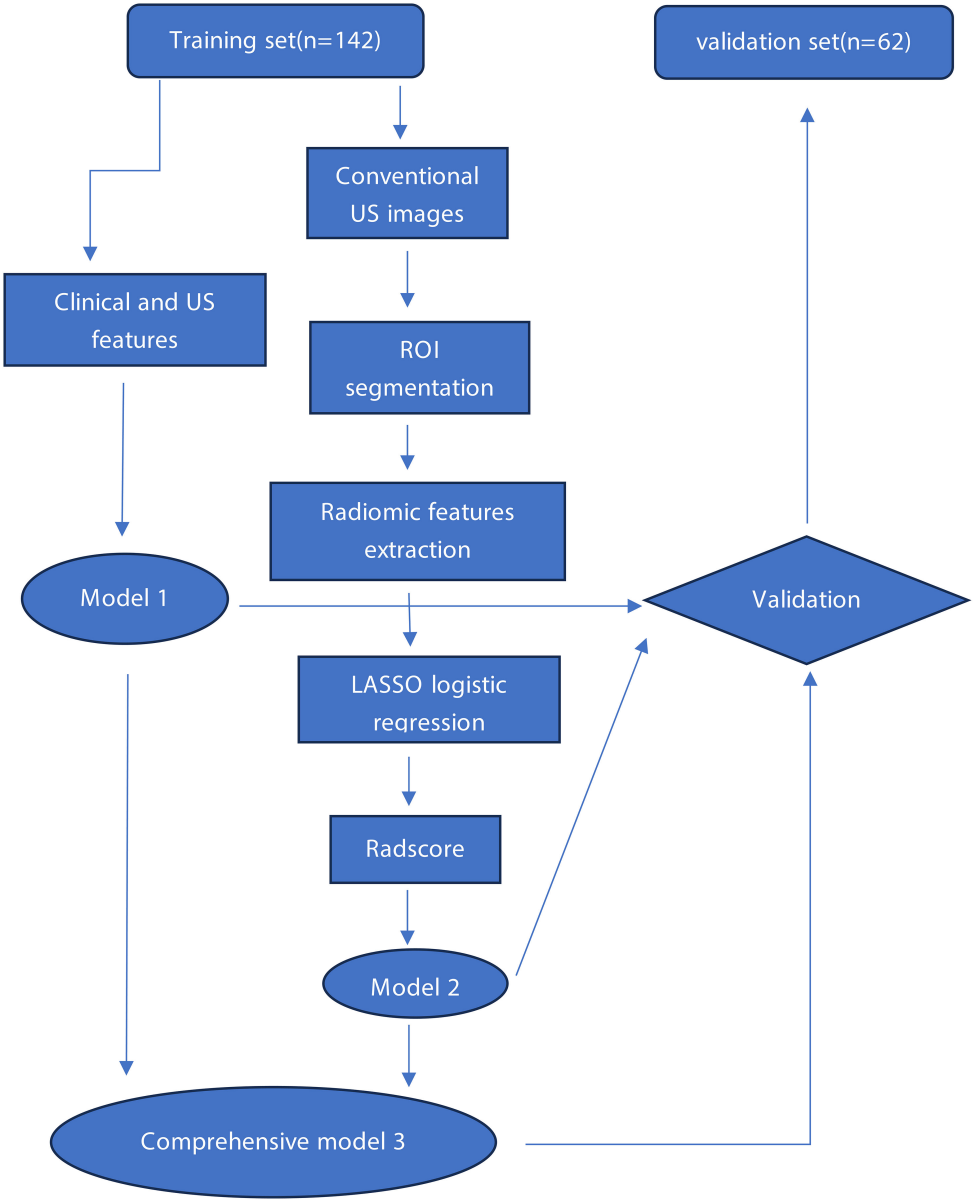
Working flowchart.

The study flowchart is shown in **Figure 1**. A total of 142 patients with a total of 142 thyroid nodules were enrolled in the training set with an average age of 42.25 ± 10.93 years (range, 24–73 years), including 41 men and 101 women. In addition, 62 patients were enrolled in the validation set with an average age of 41.61 ± 11.15 years (range, 25–70 years), including 19 men and 43 women. The proportion of lesions less than 1 cm is 72.5% (148/204), and the proportion of lesions equal 1 cm is 27.5% (56/204). The portion of surgery of central cervical lymph lode is 94.1% (192/204), and the portion of FNAB is 5.9% (12/204). The clinical and US characteristics of the training set and the validation set were summarized in **Table 1**. There was no significant difference between the two sets in clinical and US features.

**Table 1:** Univariate analysis of clinical and US features of cN0 PTMCs.

Features	Training set(67/75) Non-metastatic/metastatic lymph nodes	*p*-value	Validation set(35/27) Non-metastatic/metastatic lymph nodes	*p*-value	*p*-value^*^
**Age(years)** Mean ± SD	43.6±11.5/41.0±10.3	0.160	42.9±11.6/39.9±10.6	0.299	0.705
**Gender** Male Female	12/29 55/46	**0.006**	7/12 28/15	0.038	0.798
**BRAF type** Wild-type Mutant-type	6/7 61/68	0.938	4/3 31/24	0.969	0.637
**Position** Proximal ventral Central Proximal dorsal	37/35 10/8 20/32	0.272	22/13 2/4 11/10	0.139	0.707
**Extended to capsule** None Presence	27/24 40/51	0.303	14/6 21/21	0.138	0.614
**Aspect ratio** <1 =1 >1	25/30 2/5 40/40	0.524	15/9 2/4 18/14	0.436	0.427
**Echogenicity** Very hypoechoic Hypoechoic Isoechoic/Hyperechoic	12/9 54/64 1/2	0.559	8/2 26/23 1/2	0.208	0.541
**Echogenic foci** None Punctate echogenic foci Macrocalcifications	27/17 40/58 0	**0.023**	12/1 23/25 0/1	0.009	0.118
**Shape** Regular Irregular	44/17 23/58	**<0.001**	13/4 22/23	0.051	0.136
**Margin** Smooth Lobulated Ill-defined or irregular Spiculate Angled	0/4 9/14 5/11 38/35 15/11	0.105	5/1 5/6 5/6 14/11 6/3	0.520	0.134
**Blood flow** None Hypervascular Mild/moderate	21/28 0/0 46/47	0.454	10/7 1/2 24/18	0.705	0.223
**TIRADS Category** 3 4a 4b 4c 5	2/3 17/2 43/33 5/33 0/4	**<0.001**	2/0 14/2 17/22 2/3 0/0	0.012	0.349

* P-value comparing the two dataset cohorts.

### Model 1: clinical features and US features

**Figure 2:**
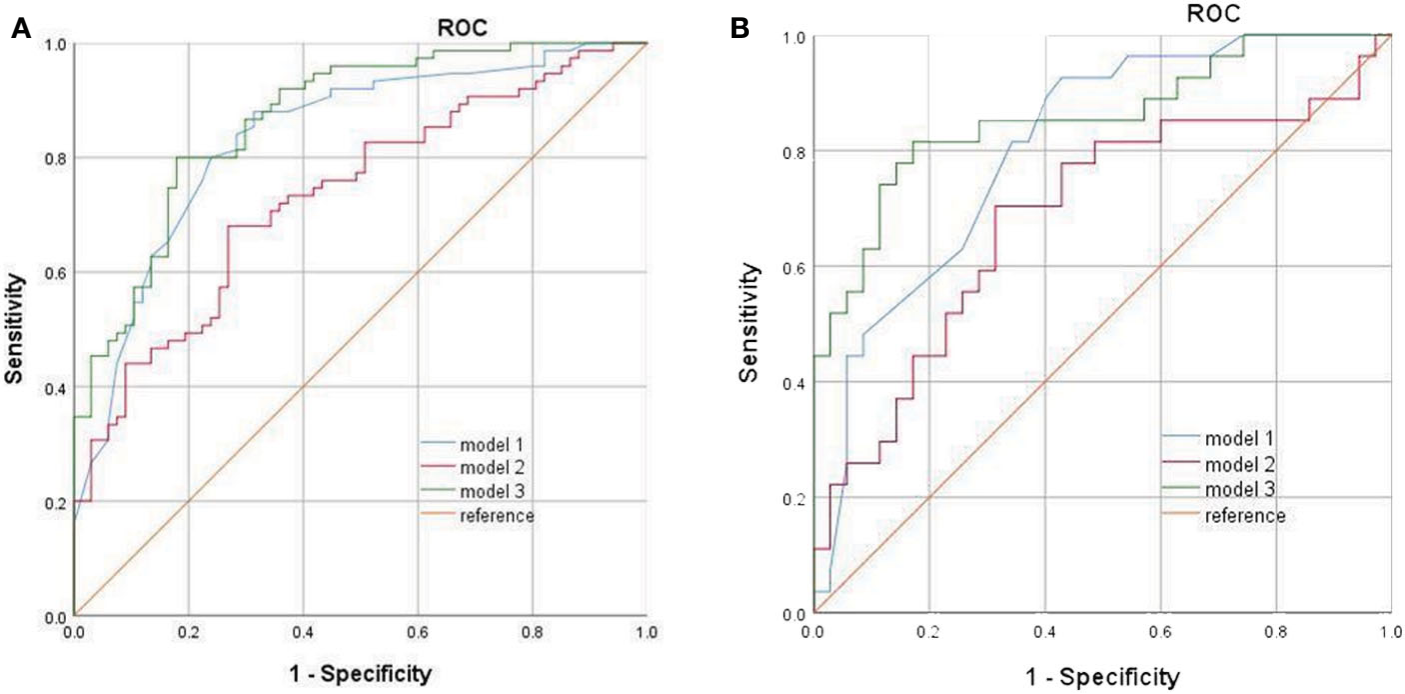
**(A)** ROCs of the training set. **(B)** ROCs of the validation set.

In the training set, univariate analysis in **Table 1** showed that four variables were related to CLNM. Model 1, constructed by combining the significant four features with ES, shows a good performance with an AUC of 0.835 (95% CI, 0.768–0.902) (**Figure 2**). The sensitivity and specificity rates were 88% and 68.7%, respectively.

### Model 2: radiomic signature

After inter and intra-observer reliability analysis and LASSO logistic regression, finally, six radiomic features was selected to build a radiomic signature. Radscore=0.421+1.29×A−2.91×B+1.56×C+8.48×D+6.76×E−6.52×F . The variables A to F represent six selected features were displayed in **Table 2**. The discriminative ability of model 2 was low with an AUC of 0.734 (95% CI, 0.653–0.815) (**Figure 2**). The sensitivity and specificity rates were 68% and 73.1%, respectively.

**Table 2:** The variables A to F and the represented the six selected radiomic features.

	US radiomic feature in formula	Lasso weighted coefficient
A	original_shape2D_Elongation	1.29
B	original_firstorder_Kurtosis	−2.91
C	wavelet.HL_glszm_GrayLevelNonUniformity	1.56
D	wavelet.HL_ngtdm_Contras	8.48
E	wavelet.HH_glszm_GrayLevelNonUniformity	6.76
F	wavelet.LL_gldm_LargeDependenceLowGrayLevelEmphasis	−6.52

### Model 3: comprehensive model

Based on the features with statistical significance, ES and radscore, a comprehensive model was built, showing an improved diagnostic efficiency with an AUC of 0.868 (95% CI, 0.811–0.925) (**Figure 2**). The sensitivity and specificity rates were 80% and 82.1%, respectively.

### The performance of different models to predict CLNM

Independent validation dataset was employed to validate the established models. In the validation set, the AUC, sensitivity, and specificity rates of model 1 were 0.806 (95% CI, 0.699–0.914), 92.6%, and 57.1%, respectively (**Figure 2**). The AUC, sensitivity, and specificity rates of model 2 were 0.686 (95% CI, 0.547–0.824), 70.4%, and 68.6%, respectively (**Figure 2**). The AUC, sensitivity, and specificity rates of model 3 were 0.857 (95% CI, 0.759–0.955), 81.5%, and 82.9%, respectively (**Figure 2**). The AUC was significantly higher in the comprehensive model than model 1 and model 2, which was shown in both the training set and the validation set(in the training set: model 3 versus model 1, p = 0.041; model 3 versus model 2, p< 0.001; in the validation set: model 3 versus model 1, p = 0.018; model 3 versus model 2, p = 009). Specificity was improved when USE and radiomic were combined.

### Performance of the nomogram (visualization of model 3)

**Figure 3:**
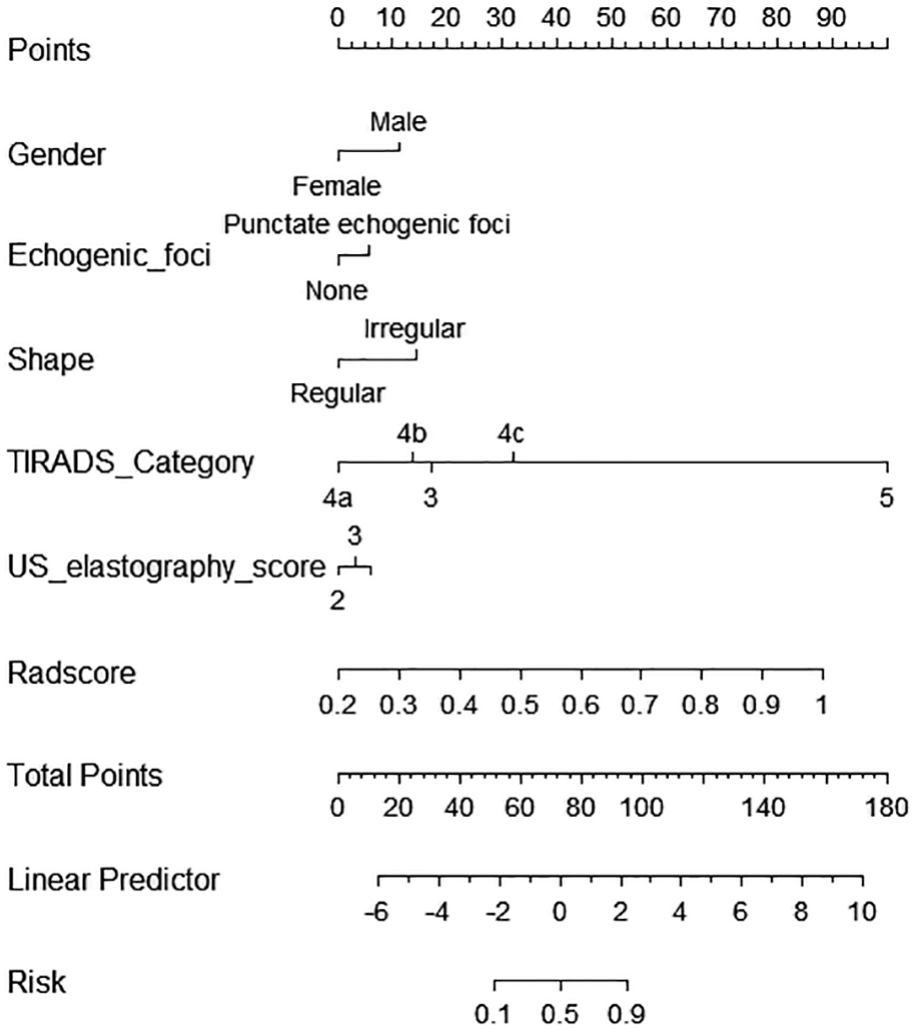
A nomogram, the visualization of the comprehensive model 3.

**Figure 4:**
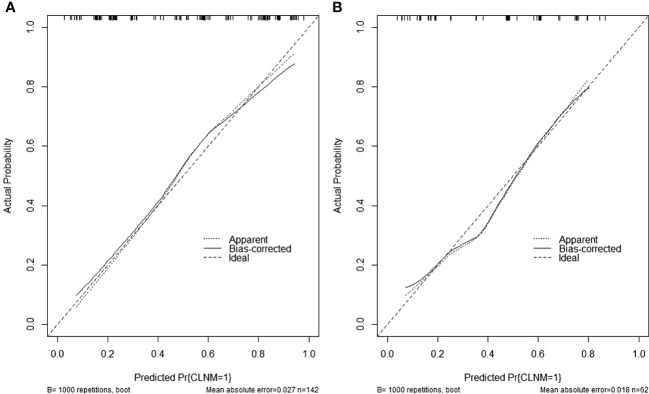
**(A)** Calibration curves of the nomogram in the training set. **(B)** Calibration curves of the nomogram in the validation set.

**Figure 5:**
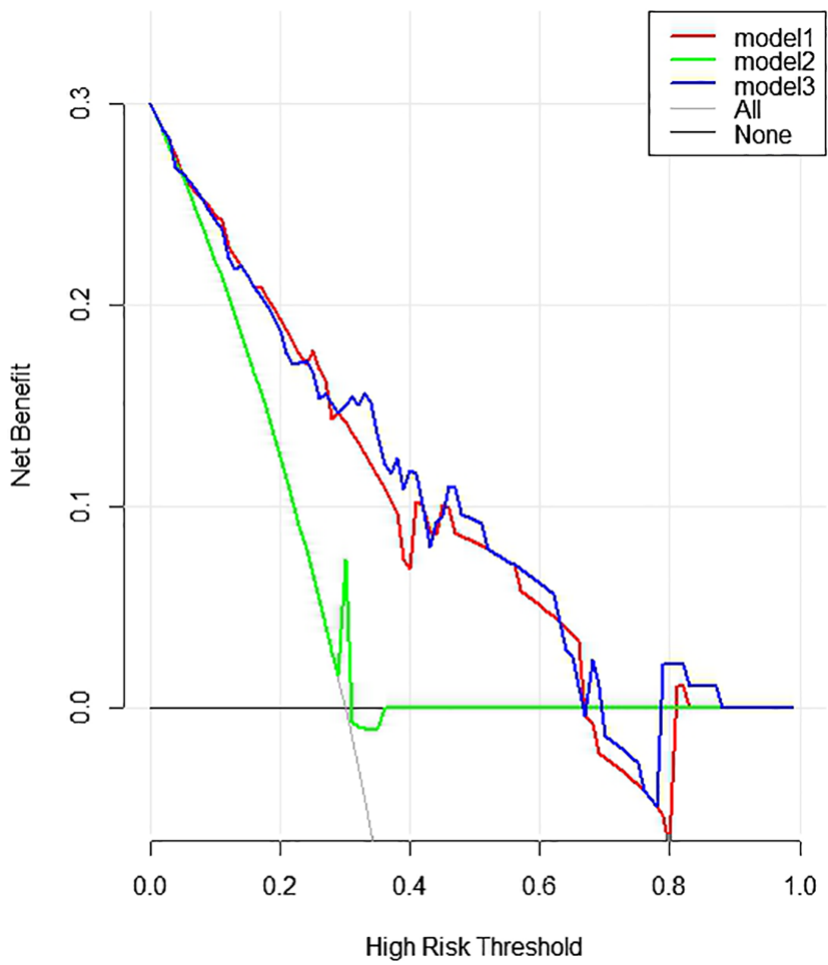
The decision curve analysis (DCA) curve evaluated the clinical value of the nomogram.

The comprehensive model (model 3) was presented as a form of nomogram (**Figure 3**). Favorable calibration curves of the nomogram were confirmed in both training and validation sets (**Figure 4**). As is shown in **Figure 5**, model 3 provided a better net benefit to predict CLNM in patients with PTMC than model 1 and model 2 for all threshold probabilities.

## Discussion

The guidelines of the American Thyroid Association suggested that active surveillance rather than surgery could be taken for low-risk PTMC. Unexpectedly, some PTMCs still progressed during surveillance. As a result, there remains debates on the value of CLND for PTMC (1). In clinical work, we are supposed to take all probabilities into account when considering which of the two management options, observation or surgery, is better or more beneficial for patients with PTMC. Thus, careful assessment of thyroid nodule and determination of risk factors related to CLNM could guide patients with PTMC to adopt appropriate management.

In our study, 102 of the 204 patients with PTMC were pathologically diagnosed with CLNM, taking a proportion of 50%, which was consistent with the incidence of 31%–60.9% in previous studies (7, 8). According to our research, male gender is closely related with CLNM in PTMCs, which is consistent with numerous studies. Gui et al. previously reported that male gender was an independent risk factor for CLNM in PTMC (23). Wen et al. found that men increases about 2.92-fold CLNM risks in cN0 PTMC (9). The unhealthy behaviors, such as smoking and alcohol consumption, of men are more likely to give rise to this result (24, 25). These studies revealed that more frequent follow-up surveillance or more aggressive treatment may be considered for men with PTMC, even though there is no evidence of CLNM.

Current studies showed that ultrasound features that only include microcalcification and irregular shape were able to predict CLNM in thyroid cancer (26–28). The findings were proved by our research to predict CLNM with a *p*-value of 0.023 and<0.001 in microcalcification and irregular shape. Microcalcifications, which are mainly caused by psammoma bodies with a smaller diameter of 10 µm to ~100 μm, were frequently seen in PTMCs. Microcalcifications are deposits for calcium salts due to the proliferation of blood vessels and fibers, which reflect the rapid growth of cancer cells (29, 30). Thus, the cervical lymph nodes should be assessed more careful if microcalcifications were found in thyroid nodules by US. Previous study pointed out that irregular tumor shape is a risk factor for multifocality and bilaterality, which are common features of PTMC and related to disease recurrence and poorer prognosis (31). Our study found that irregular shape may affect the outcome of patients with PTMC. As Kaliszewski et al. suggested, cases without clinically evident LNM but with an irregular shape should also be treated as high-risk PTMCs or symptomatic PTMCs (32). Our findings make us to agree with this view. According to Kwak-TIRADS (22), suspicious malignant features include solid component, hypoechogenicity, marked hypoechogenicity, microlobulated or irregular margins, microcalcifications, and taller-than-wide shape. As the number of suspicious US features increased, the probability of malignancy increased. Our study demonstrated that the higher risk level a thyroid nodule has, the greater possibility of CLNM a patient with PTMC may have.

USE has been applied to predict CLNM in thyroid carcinoma in recent years, and satisfactory results have been achieved. Wang et al. used SWE and calculated elasticity parameters, including Emin, Emean, and Emax of the thyroid nodule by the system to draw a conclusion that Emax ≥ 48.4 was an independent risk predictor for CLNM in PTC (33). Woo et al. also found that quantitative SWE could predict pathologic prognostic factors of LN metastasis of PTC (12). The results obtained by SWE proved that the stiffness of thyroid nodule has a positive correlation with CLNM in PTC, which made us to state that SE would have the same effect in predicting CLNM in PTMC. Disappointingly, the final visualization of model 3 (nomogram) revealed that the ES takes a small proportion to predict CLNM. Xu et al. found that conventional SE was not helpful in predicting CLNM (34), consisting with the result that higher ES was not associated with CLNM (16). Compared with the quantitative data of SWE, ES mostly relies on subjective evaluation, which may make some errors and lead to the present result. Besides, the depth of the lesions makes a difference to the score of the elastic image. Ultrasound beams are usually focused at a depth of around 3 cm to 5 cm, so that the area of maximum radiation force energy is 4 cm to 4.5 cm from the transducer and gradually diminishes as it progresses in the medium (35). If ultrasound beams reach a point beyond reference range, then their intensity is too weak to generate an adequate acoustic radiation force (36). This may result in low signal-to-noise reflecting the accuracy of ES.

As a way of machine learning, radiomic needs mass of data and the consistency of image data between the training and validation sets. By extracting a large number of features that cannot be identified by the naked eyes, more information about the tumor is mined (37, 38). In our study, the finally chosen features include one shape feature, one first-order feature, and four texture features, showing the heterogeneity of thyroid nodule comprehensively. The original_shape2D_Elongation shows the relationship between the two largest principal components in the ROI shape. Original_firstorder_Kurtosis measures the peakedness of the distribution of values in the image ROI. Wavelet.HL_glszm_GrayLevelNonUniformity and wavelet.HH_glszm_GrayLevelNonUniformity measure the variability of gray-level intensity values in the image. Wavelet.HL_ngtdm_Contras not only is a measure of the spatial intensity change but also is dependent on the overall gray level dynamic range. Wavelet.LL_gldm_LargeDependenceLowGrayLevelEmphasis measures the joint distribution of large dependence with lower gray-level values. Although model 2 did not achieve a better discrimination than model 1, an incremental improvement was obtained by incorporating the radiomic features into clinical features. The performance of model 3 was more preferable than model 1 and model 2 both in the training set and the validation set, with specificity improved significantly, making up the deficiency of ultrasound only. The luminescent spot of our study is the construction of a nomogram, which is a visualization of model 3, facilitating clinical decisions. Physicians can derive the CLNM possibility of a patient with PTMC by calculating a score for each risk factor.

Although the results of this study were promising, there were several limitations in our study. First, it was a retrospective study conducted in one institution; thus, there may exist a selection bias. In the future, a prospective research will be taken for a more accurate assessment of the prediction of CLNM in PTMCs. Second, this study is lack of an external validation data, we aim to carry out multicenter research and increase the sample size to better evaluate the clinical use of model 3. Finally, our radiomic features were only extracted from conventional US images. We will attempt to extract features from images of different modes of US, including USE and contrast-enhanced US, to dig up more information about the PTMC.

## Conclusion

The combined model based on USE and radiomic showed a superior performance in the prediction of CLNM of patients with PTMC. A nomogram based on combined model is a useful tool for clinicians to make individualized treatment strategy.

## Data availability statement

The original contributions presented in the study are included in the article/supplementary material. Further inquiries can be directed to the corresponding authors.

## Ethics statement

The studies involving humans were approved by the Ethics Committee in the First Affiliated Hospital of Nanjing Medical University. The studies were conducted in accordance with the local legislation and institutional requirements. The human samples used in this study were acquired from a by- product of routine care or industry. Written informed consent for participation was not required from the participants or the participants’ legal guardians/next of kin in accordance with the national legislation and institutional requirements.

## Author contributions

LW: Writing – original draft. YZ: Conceptualization, Data curation, Formal analysis, Methodology, Writing – original draft. LL: Formal analysis, Methodology, Writing – review & editing. WM: Conceptualization, Supervision, Writing – review & editing. HD: Writing – review & editing. XY: Writing – review & editing.

## References

[1] Haugen BR Alexander EK Bible KC Doherty GM Mandel SJ Nikiforov YE. 2015 American thyroid association management guidelines for adult patients with thyroid nodules and differentiated thyroid cancer: the american thyroid association guidelines task force on thyroid nodules and differentiated thyroid cancer. Thyroid. (2016) 26:1–133. doi: 10.1089/thy.2015.0020 26462967 PMC4739132

[2] Vaccarella S Franceschi S Bray F Wild CP Plummer M Dal Maso L. Worldwide thyroid-cancer epidemic? The increasing impact of overdiagnosis. N. Engl J Med. (2016) 375:614–7. doi: 10.1056/NEJMp1604412 27532827

[3] Davies L Morris LG Haymart M Chen AY Goldenberg D Morris J. American association of clinical endocrinologist and American college of endocrinology disease state clinical review: The increasing incidence of thyroid cancer. Endocr Pract. (2015) 21:686–96. doi: 10.4158/EP14466.DSCR PMC4923940 26135963

[4] Mehanna H Al-Maqbili T Carter B Martin E Campain N Watkinson J. Differences in the recurrence and mortality outcomes rates of incidental and nonincidental papillary thyroid microcarcinoma: a systematic review and meta-analysis of 21 329 person-years of follow-up. J Clin Endocr Metab. (2014) 99:2834–43. doi: 10.1210/jc.2013-2118 24828487

[5] Chen L Zhu Y Zheng K Zhang H Guo H Zhang L. The presence of cancerous nodules in lymph nodes is a novel indicator of distant metastasis and poor survival in patients with papillary thyroid carcinoma. J Cancer Res Clin. (2017) 143:1035–42. doi: 10.1007/s00432-017-2345-2 PMC11819005 28204971

[6] Feng JW Ye J Wu WX Qu Z Qin AC Jiang Y. Management of cN0 papillary thyroid microcarcinoma patients according to risk-scoring model for central lymph node metastasis and predictors of recurrence. J Endocrinol Invest. (2020) 43:1807–17. doi: 10.1007/s40618-020-01326-1 32557354

[7] Wada N Duh QY Sugino K Iwasaki H Kameyama K Mimura T. Lymph node metastasis from 259 papillary thyroid microcarcinomas: frequency, pattern of occurrence and recurrence, and optimal strategy for neck dissection. Ann Surg. (2003) 237:399–407. doi: 10.1097/01.SLA.0000055273.58908.19 12616125 PMC1514312

[8] Lim YC Choi EC Yoon YH Kim EH Koo BS. Central lymph node metastases in unilateral papillary thyroid microcarcinoma. Brit J Surg. (2009) 96:253–7. doi: 10.1002/bjs.6484 19224514

[9] Wen X Jin Q Cen X Qiu M Wu Z. Clinicopathologic predictors of central lymph node metastases in clinical node-negative papillary thyroid microcarcinoma: a systematic review and meta-analysis. World J Surg Oncol. (2022) 20:106. doi: 10.1186/s12957-022-02573-7 35365171 PMC8976349

[10] Yu Y Yu Z Li M Wang Y Yan C Fan J. Model development to predict central lymph node metastasis in cN0 papillary thyroid microcarcinoma by machine learning. Ann Transl Med. (2022) 10:892. doi: 10.21037/atm 36111037 PMC9469161

[11] Sigrist R Liau J Kaffas AE Chammas MC Willmann JK. Ultrasound elastography: review of techniques and clinical applications. Theranostics. (2017) 7:1303–29. doi: 10.7150/thno.18650 PMC5399595 28435467

[12] Jung WS Kim JA Son EJ Youk JH Park CS. Shear wave elastography in evaluation of cervical lymph node metastasis of papillary thyroid carcinoma: elasticity index as a prognostic implication. Ann Surg Oncol. (2015) 22:111–6. doi: 10.1245/s10434-014-3627-4 24740830

[13] Park AY Kim JA Son EJ Youk JH. Shear-wave elastography for papillary thyroid carcinoma can improve prediction of cervical lymph node metastasis. Ann Surg Oncol. (2016) 23:722–9. doi: 10.1245/s10434-016-5572-x 27654109

[14] Asteria C Giovanardi A Pizzocaro A Cozzaglio L Morabito A Somalvico F. US-elastography in the differential diagnosis of benign and Malignant thyroid nodules. Thyroid. (2008) 18:523–31. doi: 10.1089/thy.2007.0323 18466077

[15] Jin ZQ Lin MY Hu WH Li WY Bai SJ. Gray-scale ultrasonography combined with elastography imaging for the evaluation of papillary thyroid microcarcinoma: as a prognostic clinicopathology factor. Ultrasound Med Biol. (2014) 40:1769–77. doi: 10.1016/j.ultrasmedbio.2014.02.015 24768485

[16] Moon HJ Kim EK Yoon JH Kwak JY. Clinical implication of elastography as a prognostic factor of papillary thyroid microcarcinoma. Ann Surg Oncol. (2012) 19:2279–87. doi: 10.1245/s10434-011-2212-3 22246427

[17] Huang Y Zhou H Zhang C Hong Y Ye Q Huang P. Diagnostic performance of ultrasound strain elastography in transverse and longitudinal views in predicting Malignant thyroid nodules. Ultrasound Med Biol. (2019) 45:2289–97. doi: 10.1016/j.ultrasmedbio.2019.05.018 31196745

[18] Tong Y Li J Huang Y Zhou J Liu T Guo Y. Ultrasound-based radiomic nomogram for predicting lateral cervical lymph node metastasis in papillary thyroid carcinoma. Acad Radiol. (2021) 28:1675–84. doi: 10.1016/j.acra.2020.07.017 32782219

[19] Liu T Ge X Yu J Guo Y Wang Y Wang W. Comparison of the application of B-mode and strain elastography ultrasound in the estimation of lymph node metastasis of papillary thyroid carcinoma based on a radiomics approach. Int J Comput Ass Rad. (2018) 13:1617–27. doi: 10.1007/s11548-018-1796-5 29931410

[20] Peng Y Zhang ZT Wang TT Wang Y Li CH Zuo MJ. Prediction of central lymph node metastasis in cN0 papillary thyroid carcinoma by CT radiomics. Acad Radiol. (2023) 30(7):1400–7. doi: 10.1016/j.acra.2022.09.002 36220726

[21] Lu W Zhong L Dong D Fang M Dai Q Leng S. Radiomic analysis for preoperative prediction of cervical lymph node metastasis in patients with papillary thyroid carcinoma. Eur J Radiol. (2019) 118:231–8. doi: 10.1016/j.ejrad.2019.07.018 31439247

[22] Kwak JY Han KH Yoon JH Moon HJ Son EJ Park SH. Thyroid imaging reporting and data system for US features of nodules: a step in establishing better stratification of cancer risk. Radiology. (2011) 260:892–9. doi: 10.1148/radiol.11110206 21771959

[23] Gui CY Qiu SL Peng ZH Wang M. Clinical and pathologic predictors of central lymph node metastasis in papillary thyroid microcarcinoma: a retrospective cohort study. J Endocrinol Invest. (2018) 41:403–9. doi: 10.1007/s40618-017-0759-y 28884301

[24] Sun W Lan X Zhang H Wang M. Risk factors for central lymph node metastasis in CN0 papillary thyroid carcinoma: A systematic review and meta-analysis. PloS One. (2015) 10:e139021. doi: 10.1371/journal.pone.0139021 PMC4592212 26431346

[25] Xu SY Yao JJ Zhou W Chen L Zhan WW. Clinical characteristics and ultrasonographic features for predicting central lymph node metastasis in clinically node-negative papillary thyroid carcinoma without capsule invasion. Head Neck J Sci Spec. (2019) 41:3984–91. doi: 10.1002/hed.25941 31463972

[26] Li F Pan D He Y Wu Y Peng J Li J. Using ultrasound features and radiomics analysis to predict lymph node metastasis in patients with thyroid cancer. BMC Surg. (2020) 20:315. doi: 10.1186/s12893-020-00974-7 33276765 PMC7716434

[27] Liu J Zheng D Li Q Tang X Luo Z Yuan Z. A predictive model of thyroid Malignancy using clinical, biochemical and sonographic parameters for patients in a multi-center setting. BMC Endocr Disord. (2018) 18:17. doi: 10.1186/s12902-018-0241-7 29514621 PMC5842594

[28] Watanabe K Igarashi T Ashida H Ogiwara S Ohta T Uchiyama M. Diagnostic value of ultrasonography and TI-201/Tc-99m dual scintigraphy in differentiating between benign and Malignant thyroid nodules. Endocrine. (2019) 63:301–9. doi: 10.1007/s12020-018-1768-0 30276595

[29] Gao X Luo W He L Cheng J Yang L. Predictors and a prediction model for central cervical lymph node metastasis in papillary thyroid carcinoma (cN0). Front Endocrinol. (2021) 12:789310. doi: 10.3389/fendo.2021.789310 PMC8828537 35154002

[30] Li X Zhou W Zhan W. Clinical and ultrasonographic features of medullary thyroid microcarcinomas compared with papillary thyroid microcarcinomas: a retrospective analysis. BMC Med Imaging. (2020) 20:49. doi: 10.1186/s12880-020-00444-9 32410587 PMC7227110

[31] Kaliszewski K Diakowska D Wojtczak B Migoń J Kasprzyk A Rudnicki J. The occurrence of and predictive factors for multifocality and bilaterality in patients with papillary thyroid microcarcinoma. Medicine. (2019) 98:e15609. doi: 10.1097/MD.0000000000015609 31083255 PMC6531220

[32] Kaliszewski K Diakowska D Rzeszutko M Nowak Ł Aporowicz M Wojtczak B. Risk factors of papillary thyroid microcarcinoma that predispose patients to local recurrence. PloS One. (2020) 15:e244930. doi: 10.1371/journal.pone.0244930 PMC7775061 33382852

[33] Wang B Cao Q Cui XW Dietrich CF Yi AJ. A model based on clinical data and multi-modal ultrasound for predicting cervical lymph node metastasis in patients with thyroid papillary carcinoma. Front Endocrinol. (2022) 13:1063998. doi: 10.3389/fendo.2022.1063998 PMC9791085 36578956

[34] Xu JM Xu XH Xu HX Zhang YF Guo LH Liu LN. Prediction of cervical lymph node metastasis in patients with papillary thyroid cancer using combined conventional ultrasound, strain elastography, and acoustic radiation force impulse (ARFI) elastography. Eur Radiol. (2016) 26:2611–22. doi: 10.1007/s00330-015-4088-2 26560715

[35] Barr RG Ferraioli G Palmeri ML Goodman ZD Garcia-Tsao G Rubin J. Elastography assessment of liver fibrosis: society of radiologists in ultrasound consensus conference statement. Radiology. (2015) 276:845–61. doi: 10.1148/radiol.2015150619 26079489

[36] Bouchet P Gennisson J Podda A Alilet M Carrié M Aubry S. Artifacts and technical restrictions in 2D shear wave elastography. Ultraschall der Med (Stuttgart Germany: 1980). (2020) 41:267–77. doi: 10.1055/a-0805-1099 30577047

[37] Gillies RJ Kinahan PE Hricak H. Radiomics: images are more than pictures, they are data. Radiology. (2016) 278:563–77. doi: 10.1148/radiol.2015151169 PMC4734157 26579733

[38] Lambin P Rios-Velazquez E Leijenaar R Carvalho S van Stiphout RG Granton P. Radiomics: extracting more information from medical images using advanced feature analysis. Eur J Cancer (Oxford England: 1990). (2012) 48:441–6. doi: 10.1016/j.ejca.2011.11.036 PMC4533986 22257792

